# Maternal sevoflurane exposure increases the epilepsy susceptibility of adolescent offspring by interrupting interneuron development

**DOI:** 10.1186/s12916-023-03210-0

**Published:** 2023-12-21

**Authors:** Xinyue Liang, Ming Jiang, Hao Xu, Tianxiang Tang, Xiangpeng Shi, Yi Dong, Lei Xiao, Yunli Xie, Fang Fang, Jing Cang

**Affiliations:** 1 Department of Anesthesia, Zhongshan Hospital, Fudan University, Shanghai, China; 2 State Key Laboratory of Medical Neurobiology and MOE Frontiers Center for Brain Science, Institutes of Brain Science, Fudan University, Shanghai, China; 3 Key Laboratory of Adolescent Health Assessment and Exercise Intervention of Ministry of Education, East China Normal University, Shanghai, China

**Keywords:** Anesthetic neurotoxicity, Epilepsy susceptibility, Interneuron tangential migration, CXCL12/CXCR4 pathway

## Abstract

**Background:**

Exposure to general anesthesia influences neuronal functions during brain development. Recently, interneurons were found to be involved in developmental neurotoxicity by anesthetic exposure. But the underlying mechanism and long-term consequences remain elusive.

**Methods:**

Pregnant mice received 2.5% sevoflurane for 6-h on gestational day 14.5. Pentylenetetrazole (PTZ)-induced seizure, anxiety- and depression-like behavior tests were performed in 30- and 60-day-old male offspring. Cortical interneurons were labeled using Rosa26-EYFP/-; Nkx2.1-Cre mice. Immunofluorescence and electrophysiology were performed to determine the cortical interneuron properties. Q-PCR and in situ hybridization (ISH) were performed for the potential mechanism, and the finding was further validated by in utero electroporation (IUE).

**Results:**

In this study, we found that maternal sevoflurane exposure increased epilepsy susceptibility by using pentylenetetrazole (PTZ) induced-kindling models and enhanced anxiety- and depression-like behaviors in adolescent offspring. After sevoflurane exposure, the highly ordered cortical interneuron migration was disrupted in the fetal cortex. In addition, the resting membrane potentials of fast-spiking interneurons in the sevoflurane-treated group were more hyperpolarized in adolescence accompanied by an increase in inhibitory synapses. Both q-PCR and ISH indicated that CXCL12/CXCR4 signaling pathway downregulation might be a potential mechanism under sevoflurane developmental neurotoxicity which was further confirmed by IUE and behavioral tests. Although the above effects were obvious in adolescence, they did not persist into adulthood.

**Conclusions:**

Our findings demonstrate that maternal anesthesia impairs interneuron migration through the CXCL12/CXCR4 signaling pathway, and influences the interneuron properties, leading to the increased epilepsy susceptibility in adolescent offspring. Our study provides a novel perspective on the developmental neurotoxicity of the mechanistic link between maternal use of general anesthesia and increased susceptibility to epilepsy.

**Supplementary Information:**

The online version contains supplementary material available at 10.1186/s12916-023-03210-0.

## Background

Every year, approximately 0.75–2% of pregnant women need non-obstetric surgery under general anesthesia to treat various medical conditions, such as appendicitis, cholecystitis, gynecological malignancy, or traumatic injuries [1]. Middle pregnancy (namely, the second trimester) is considered a safe period for surgical anesthesia, but is also the crucial period for fetal neurogenesis. Numerous studies have indicated a link between prolonged maternal exposure to anesthesia and neuronal deficits in offspring, but the underlying mechanisms remain elusive [2].

Fetal neurogenesis is exquisitely sensitive to environmental toxins or chemicals, including general anesthetics [3]. Studies in rodents have reported that maternal anesthesia exposure inhibited the migration of pyramidal neurons and caused behavioral deficits and cognitive defects in young offspring [4–6]. Recently, interneurons were also found to be involved in developmental neurotoxicity due to repeated propofol exposure, resulting in motor learning impairment in adulthood [7]. However, to date, few studies have directly focused on the influence of anesthesia on interneuron development and its long-term consequences.

In mice, cortical neurons consist of γ-aminobutyric acid (GABA)-ergic inhibitory interneurons (~20%) and glutamatergic excitatory pyramidal neurons (∼80%); the former are the main source of inhibition in the neocortex [8]. To maintain cortical function, a balance between excitatory and inhibitory neurons is critical. Pyramidal neurons are generated in the proliferative zones of the dorsal telencephalon and radially migrate towards the pial surface [9]. The majority of cortical interneurons originate from the subcortical areas of the ventral telencephalon and reach the cortex by moving along tangential paths, with a peak at embryonic day (E) 13.5–16.5 in rodents [10]. At the end of the tangential migration, interneurons depart from the tangential migration paths, commence radial migration, and settle in appropriate cortical laminae [11]. During this process, interneurons precisely integrate cell-intrinsic characteristics according to input from local environmental cues to facilitate appropriate migratory patterns [11]. The major source of cortical interneurons is the medial ganglionic eminence (MGE), which generates ~70% of all cortical interneurons in rodents. MGE-derived interneurons include two major classes: fast-spiking interneurons that express the calcium buffer parvalbumin (PV) and non-fast-spiking interneurons that express the neuropeptide somatostatin (SST) [11].

Appropriate interneuron migration and distribution are essential for the construction of functional neuronal circuitry and the maintenance of excitatory/inhibitory (E/I) balance in the brain [12]. Any disturbance of these processes may lead to postnatal interneuron dysfunction and E/I imbalance in neural circuits [12]. Moreover, accumulating evidence shows that the loss or dysfunction of GABAergic interneurons can promote epilepsy [13]. Furthermore, interneuron-related therapies, such as grafting MGE progenitor cells [14–16], recruitment of surviving endogenous interneurons, and reprogramming reactive glia into interneurons, reduce seizures in epilepsy models [17, 18].

Recently, a large-scale retrospective study in adults showed that general anesthesia might interfere with postoperative epilepsy. After adjusting for confounding factors, they found a significant association between general anesthesia and the one-year incidence of postoperative epilepsy in individuals 20–39 years of age [19]. As the development of neural circuits in the brain continues until 20–25 years of age in humans [20], the increased epilepsy susceptibility after general anesthesia may be associated with impaired neural circuits. In addition, in juvenile patients, epilepsy is always associated with brain malformations during development [21]. Thus, it is highly important to determine the effect of anesthesia exposure during early neural development on epilepsy susceptibility.

In this study, we assessed the effect of maternal sevoflurane exposure on epilepsy susceptibility and anxiety- and depression-like behaviors in offspring. We hypothesized that sevoflurane, the most commonly used anesthetic in pregnant women, would impair the migration of MGE-derived interneurons in the embryonic cortex in mice and increase epilepsy susceptibility and abnormal behaviors in adolescent offspring.

## Methods

### Animals

All experimental animals were kept in an animal facility at Fudan University and all experiments were conducted in accordance with guidelines approved by Fudan University. C57BL/6J wild-type mice were purchased from the SLAC Laboratory, and their offspring underwent epilepsy susceptibility assays and behavioral tests. Transgenic heterozygous Rosa26-EYFP/- mice and Nkx2.1-Cre/- mice on a C57BL/6J background were crossed to generate Rosa26-EYFP/-; Nkx2.1-Cre/- mice for histological and electrophysiological assays. Animals were housed in a temperature-controlled (23°–24°C) room under a 12-h light/dark period (8:00 A.M. light ON and 8:00 P.M. light OFF). Water and standard mouse chow were available ad libitum. Male and female mice were mated in a 1:2 ratio. The day when a vaginal plug was detected was considered E0.5. The female mice with vaginal plug were single housed in a new cage.

### Animal anesthesia

According to the minimum alveolar concentration (MAC) of C57BL/6J mice determined in our previous study [22], we utilized 2.5% sevoflurane (approximately 1.0 MAC) in this study. At E14.5, the pregnant mice were randomly assigned to the control (Ctr) group, receiving 100% O_2_ exposure, or the sevoflurane-treated (Sevo) group, receiving exposure to 2.5% sevoflurane mixed in 97.5% O_2_ for 6 h. The mice in the Sevo group were anesthetized inside a 20 × 30 × 20-cm box, lined with a heating pad to prevent hypothermia. For the embryonic experiments, embryonic brains were collected at the end of O_2_/sevoflurane treatment. For the postnatal experiments, mice were returned to their cages after recovering from anesthesia.

### Pentylenetetrazol (PTZ)-induced seizures

As previously described [23], we used the convulsant agent PTZ (P6500, Sigma-Aldrich) to induce seizures in postnatal day (P) 30 and P60 male offspring mice. The mice that experienced prenatal O_2_/sevoflurane exposure were intraperitoneally injected with 12.5 mg kg^−1^ PTZ every 10 min until generalized seizures occurred. After injection, the mice were immediately returned to the test cage to record seizure activity. PTZ-induced seizure behaviors were scored on the Racine scale. The cumulative doses and latencies to induce the first minimal and tonic-clonic seizures (defined as head nodding with forelimb clonus and rearing with uncontrolled falling, respectively) were determined for each mouse by analyzing the video recording.

### Mouse behavioral tests

The male offspring from the Ctr and Sevo groups were tested at P30 and P60. For testing, the mice were transferred into the behavioral testing room at least 2 h beforehand. Behavioral experiments were conducted between 9:00 and 18:00, during the light phase of the light/dark cycle. The movements of the mice were tracked and recorded with EthoVision XT 8.5 (Noldus).

The open field test (OFT) was performed in a box (40 × 40 × 40 cm). Male mice were placed individually in the center of the box at the start and allowed to move freely for 10 min. The total distance traveled was determined to assess general locomotor activity. The duration in and number of visits to the center region during the 10 min were recorded.

The elevated plus maze (EPM) test was conducted in an elevated plus-maze that consisted of a central platform (5 cm × 5 cm) with plus-shaped arms (30 cm × 5 cm), extending on either side of it; the enclosed arm was equipped with 15-cm-high walls. The maze was elevated to a height of 60 cm above the floor. At the start, mice were individually placed on the central platform facing an open arm of the maze. The time each mouse spent in the open arms during the total 5-min period was calculated and used to assess the anxiety-like behavior.

The tail suspension test (TST) was performed by attaching the tail of a mouse to a wood rod with adhesive tape, thus suspending the mouse 35 cm above the ground. During the 5-min testing period, the duration of immobility was recorded by a stopwatch.

### Immunofluorescence

Embryonic brains were harvested by cesarean sections. Postnatal mice were anesthetized with pentobarbital, and then transcardially perfused with phosphate buffered saline (PBS) followed by 4% paraformaldehyde (PFA). The brains were fixed in 4% PFA overnight at 4°C. Then, the brains were transferred to a PBS solution containing 30% sucrose until they sank to the bottom of the tube; brains were then embedded in an OCT compound (Sakura). The embryonic and postnatal brains were coronally sectioned into 14-μm and 30-μm thick slices, respectively. After antigen recovery, cryosections were permeabilized in 0.5% Triton X-100 in PBS for 1 h and then incubated with blocking solution (0.3% Triton X-100 and 5% normal donkey serum in PBS) for 30 min at room temperature. Next, slices were incubated at 4°C overnight with primary antibodies. After thoroughly washing with PBS, slices were incubated in the dark for 2 h at room temperature with secondary antibodies. DAPI staining was performed before the slices were mounted. All the antibodies are listed in Additional file 1: Table S1.

### Image acquisition and analysis

At least three embryos or postnatal mice from different mothers were used in each group. Images were acquired by fluorescence microscopy (Nikon) or confocal microscopy (Nikon A1R) and then processed by NIS-Elements AR and ImageJ.

To analyze the quantity and distribution of yellow fluorescent protein-positive (YFP+) cells, the cortices of embryonic and postnatal mice were imaged by fluorescence microscopy with a 10× objective and analyzed in 200-μm and 800-μm sections, respectively; these sections spanned from the marginal zone (MZ) to the ventricular zone (VZ). The number of YFP+ cells in the brain of each offspring mouse was manually counted across multiple coronal sections and averaged within groups for calculation. The number of cells in each bin or layer was manually counted across multiple coronal sections in ImageJ.

The orientations of the leading processes were quantitatively analyzed as previously described [24]. To analyze the rate and the direction of interneuron migration, a line was drawn from the end of the leading process to the soma, and the angle of the line was measured [medial direction was defined as 0° (360°), ventral as 90°, lateral as 180°, dorsal as 270°].

The number of synapses around somas was measured as previously described [25]. The neurons in cortical layer (L) 5 were imaged by confocal microscopy with a 60× immersion objective. Each cell was imaged in successive 1-μm increments from the cell surface and then projected across the *Z*-axis to obtain the maximum intensity for analysis. All images were captured with the same parameters. The numbers of presynaptic (Glut1+ and GAT1+) and postsynaptic (PSD95+ and Gephyrin+) boutons within 2 μm of the soma were manually counted in ImageJ. The Glut1/GAT1 ratios were determined by ImageJ in a 50-μm × 50-μm area.

### Electrophysiological recording and analysis

Electrophysiological recording and analysis were conducted according to previously described methods [26]. The brains of P30 Rosa26-EYFP/-; Nkx2.1-Cre mice were prepared for acute brain sections. For electrophysiological recording, a slice was transferred to a recording chamber under constant perfusion with oxygenated artificial cerebral spinal fluid (ACSF) at a flow rate of 1.5–2 ml/min, with the temperature maintained at ~30°C by a feedback inline heater (TC-324C; Warner Instruments) during recording. Cortical interneurons were visualized in slices using an IR-DIC microscopy, and identified based on the YFP signal. L5 YFP+ cells were recorded using the 700B amplifier; data were digitized at 10 kHz, filtered at 4 kHz, and collected using pCLAMP software (Molecular Devices).

To assess the properties of action potentials (APs), the first neuronal spike induced by current injection was detected and analyzed. The data within 100 ms of the spike peak were used for phase plot analysis (membrane potential Vm vs. dVm/dt). According to spike and phase plot analysis, the following parameters were calculated: spike threshold, spike amplitude, halfwidth, depolarizing rate max, repolarizing rate max, spike rise time, and decay time.

### Quantitative real-time polymerase chain reaction (q-PCR)

The total RNA was isolated, and cDNA was synthesized using a HifairIII First-Strand cDNA Synthesis Kit (Yeasen). QuantStudio3 Real-Time PCR Systems (Thermo Fisher Scientific) were used for q-PCR with SYBR Green Master Mix (Yeasen). QuantStudio Design & Analysis Software (Thermo Fisher Scientific) was used for quantification, and the data were normalized to the level of GAPDH mRNA. Each sample was measured in triplicate, and the 2^−ΔΔCt^ method was used. CXCL12 and CXCR4 primer pairs are reported in Additional file 1: Table S1.

### In situ hybridization (ISH)

ISH was performed according to a published protocol [27]. Embryonic brains at E14.5 were fixed with 4% PFA overnight at 4°C and dehydrated with 30% sucrose in diethylpyrocarbonate (DEPC)-treated PBS until they sank. Then, the brains were embedded in an OCT compound and sectioned at a thickness of 20 μm. Digoxigenin (DIG) -labeled RNA probes were constructed by in vitro transcription using DIG RNA labeling mix (11277073910, Roche). Sections were incubated with probes at 65°C overnight. After washing with hybridization buffer, the sections were incubated with anti-DIG-AP (11093274910, Roche) at 4°C overnight. The AP activity was developed using NBT/BCIP substrates (Roche, 11681451001). CXCL12 ISH probe primers are reported in Additional file 1: Table S1.

### In utero electroporation (IUE)

Plasmids of pCAGEN-SBP-DICER1 (#50558) and pCAG-RFP (#89687) were purchased from Addgene. Mouse CXCL12 was amplified and cloned into the EcoRI/XhoI sites of the p3xFLAG-CMT-14 vector. CXCL12 clone primers are reported in Additional file 1: Table S1.

IUE was performed according to previously published methods [27]. Pregnant mice at E13.5 were anesthetized with isoflurane (3% isoflurane for induction and 2% isoflurane during surgery for maintenance). Mouse abdomens were cleaned with 70% ethanol. A midline incision was made, and the uterine horns were exposed. Desired plasmids (0.1 mg/mL pCAG-RFP with or without 1.5–2.0 mg/mL pCAGEN-CXCL12) diluted in 1 μl sterile Tris-EDTA buffer (pH 7.4), which contained Fast Green (Sigma-Aldrich), were injected into the lateral ventricle of embryos with a glass micropipette. Five 50-ms pulses of 33 V at 950-ms intervals were applied with a BTX electroporation system (ECM830). After electroporation, the uterine horns were returned inside the body, and the incision was sutured to allow the embryos to continue developing. The mouse dam was placed on a 37°C heated plate until recovery from the anesthesia. The entire surgical procedure was completed within 30 min. Embryonic brains were used for further experiments at E14.5.

### Statistical analysis

Animals were randomly assigned into treatment groups. Sample size (*n*) was indicated in the figure legends. To determine an appropriate number of animals, we estimated sample size based on our previous works and those commonly used in the field [6, 27]. All quantifications and analyses were performed by investigators blinded to treatment conditions. GraphPad Prism 8 was used for data analysis and graphical presentation. Normally distributed data were analyzed using Student’s *t* tests (for two groups) or one-way ANOVAs (for more than two groups). For simultaneous comparison of two parameters between two and more groups, we used two-way ANOVAs and Bonferroni’s post hoc tests. The corresponding statistical test is indicated in each figure. In all cases, *p* < 0.05 was considered statistically significant. Asterisks denote statistical significance (i.e., *p < 0.05; **p < 0.01; and ***p < 0.001). All data are expressed as the mean ± standard error of the mean (SEM).

## Results

### Maternal sevoflurane exposure increases the epilepsy susceptibility of adolescent offspring

**Fig. 1:**
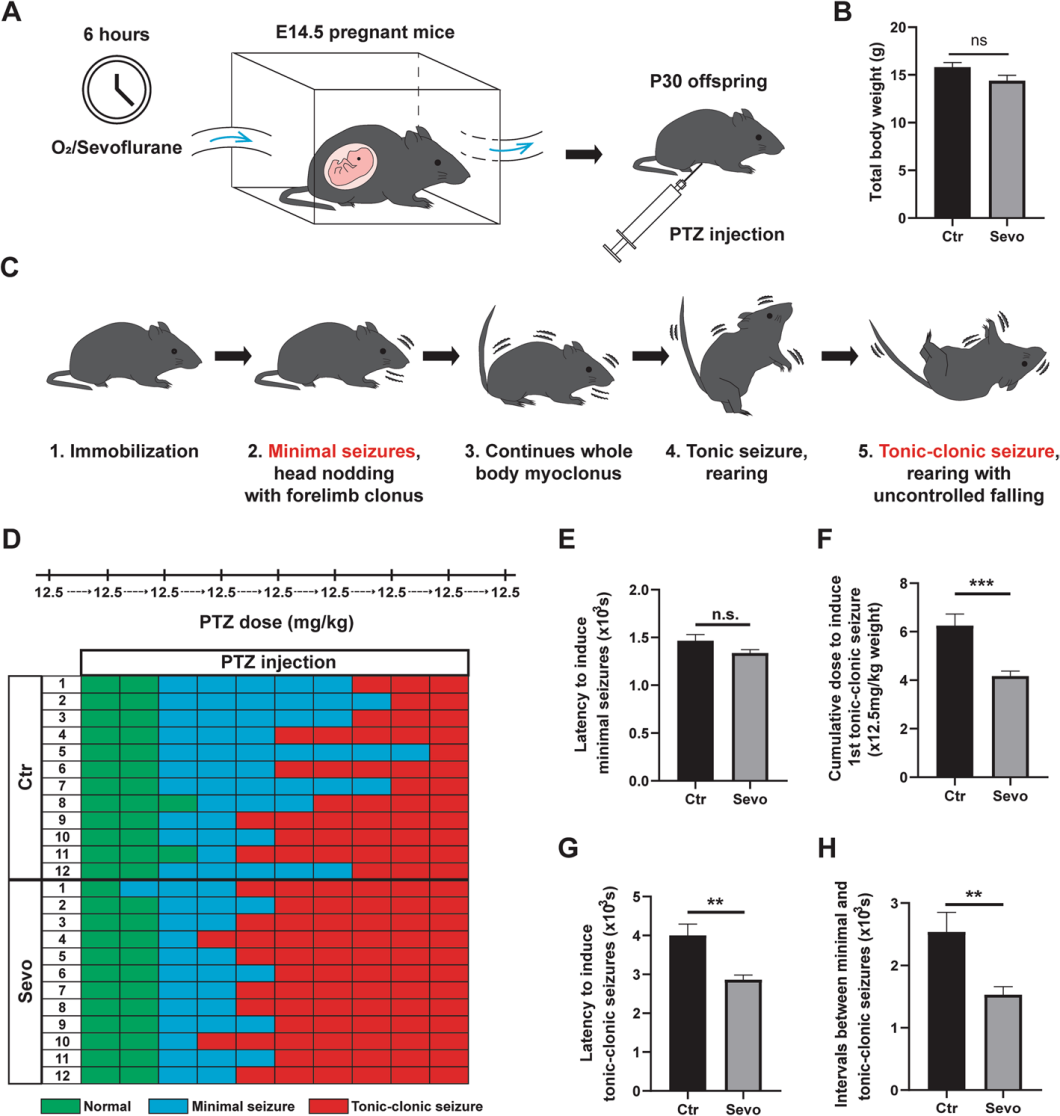
Maternal sevoflurane exposure increases the epilepsy susceptibility of adolescent offspring. **A** Experimental procedures. **B** Quantification of total body weight in the two groups. **C** Illustrations of representative mice behaviors for respective seizure scores according to the Racine scale. **D** Overall PTZ injections to induce seizures in Ctr and Sevo groups respectively. Note the lower number of PTZ injections for inducing tonic-clonic seizures in the Sevo group. **E**–**H** Comparisons about the latency to induce minimal seizures (**E**), the cumulative doses and the latencies required to induce the 1st tonic-clonic seizures (**F, G**), and the intervals between minimal and tonic-clonic seizures (**H**). The data are represented as mean ± SEM. Ctr: *n*=12; Sevo: *n*=12. Two-tailed Student’s *t*-test was performed for statistical analysis. **p < 0.01, ***p < 0.001, n.s. no significance

To investigate the association between maternal sevoflurane exposure and the epilepsy susceptibility of offspring, we used a well-established rodent model of epilepsy, in which seizures are induced by the convulsant agent pentylenetetrazol (PTZ) [23]. Pregnant mice at E14.5, corresponding to middle pregnancy in humans [28], were randomly assigned to the control (Ctr) group (O_2_ exposure for 6 h) or the sevoflurane-treated (Sevo) group (2.5% sevoflurane exposure for 6 h) (Fig. 1A). At P30, equivalent to the adolescent stage in humans [28], the total body weight had no difference between the Ctr or Sevo group (Fig. 1B), and we did not detect any spontaneous seizure activity in offspring from both groups. Subsequently, PTZ was administered to the offspring to induce seizures (Fig. 1A). Seizure behaviors were scored based on Racine’s scale (Fig. 1C, D).

We observed that the time to induce minimal seizures including head nodding and forelimb clonus, was similar in both the Ctr and Sevo groups (Fig. 1E), indicating that prenatal sevoflurane exposure did not increase epilepsy susceptibility under low-dose PTZ treatment. However, the cumulative doses of PTZ and the latency to the onset of generalized tonic-clonic seizures were significantly lower in the Sevo group than in the Ctr group (Fig. 1F, G), consistent with the significantly shortened interval between minimal and tonic-clonic seizures (Fig. 1H). These results suggest that maternal sevoflurane exposure increases epilepsy susceptibility in adolescent offspring.

### Maternal sevoflurane exposure results in abnormal behaviors in adolescent offspring

**Fig. 2:**
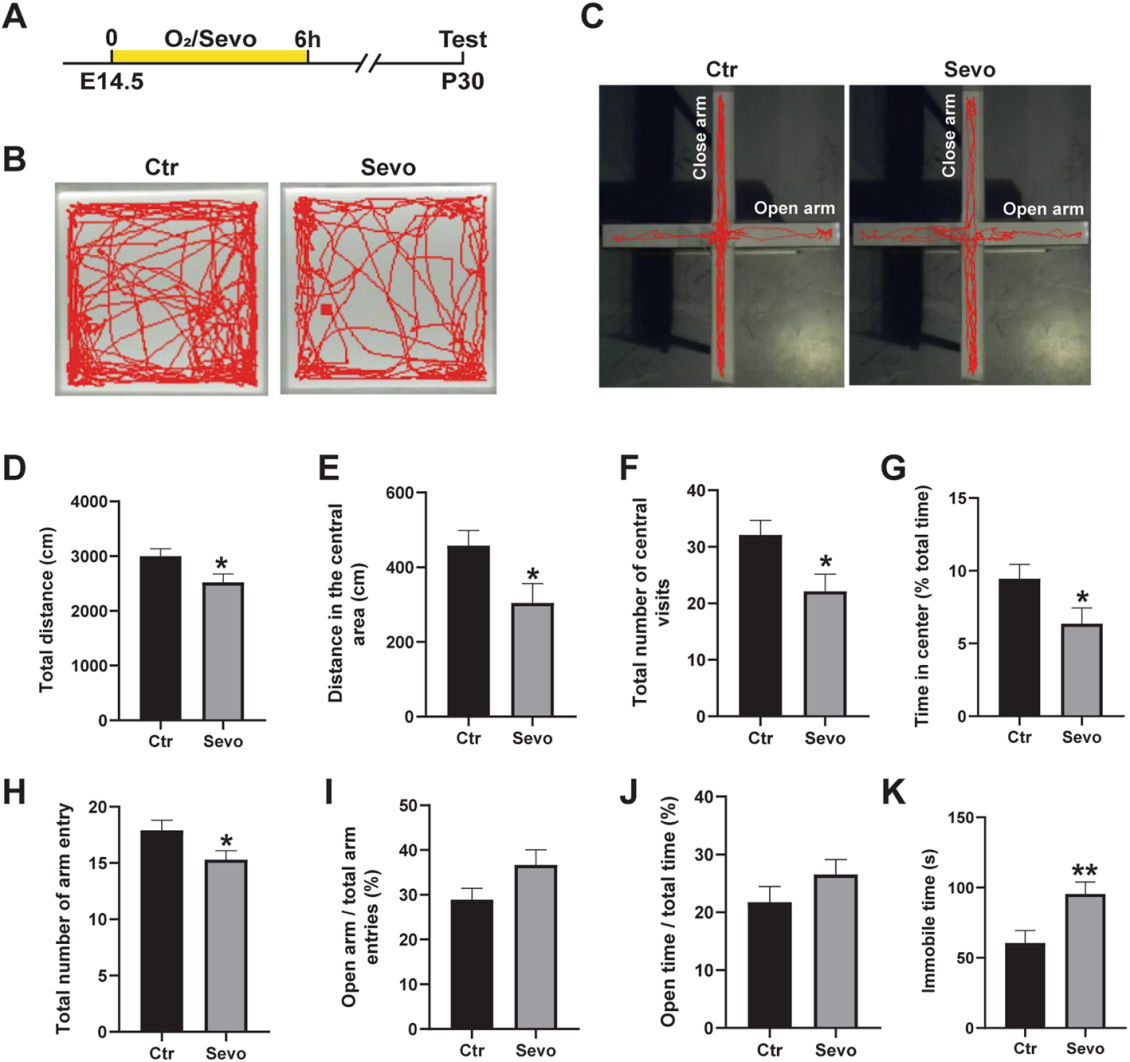
Maternal sevoflurane exposure results in abnormal behaviors in adolescent offspring. **A** Experimental procedures. **B** The representative traces in the OFT within 10 min. **C** The representative traces in the EPM in 5 min. **D**–**G** Comparisons about the total distance traveled (**D**), the central distance traveled (**E**), the total number of central visits (**F**), and the percentage of time spent in the central area (**G**), *p*=0.02390, 0234, 0.0161, and 0.0431, respectively. Ctr: *n*=20; Sevo: *n*=16. **H-J** Comparisons about the total number of arm entries (*p*=0.0429) (**H**), the ratio of the number of open arm entries and total arm entries (**I**), and the ratio of the duration in open arm and total time (**J**). Ctr: *n*=20; Sevo: *n*=16. **K** The immobile time in the TST, *p*=0.0071. Ctr: *n*=21; Sevo: *n*=19. The data are represented as mean ± SEM. Two-tailed Student’s *t*-test was performed for statistical analysis. *p < 0.05, **p < 0.01

Anxiety and depression interacting with E/I imbalance are often comorbid with epilepsy [29]. Therefore, the open field test (OFT) and elevated plus-maze (EPM) test were performed to assess anxiety-like behaviors, and the tail suspension test (TST) was used to assess depression-like behavior (Fig. 2A–C). In the OFT, the mice in the Sevo group showed a shorter total distance traveled (Fig. 2D), indicating a reduced activity relative to the Ctr group. In addition, the central distance traveled, the number of central visits, and the percentages of total exploration time in the central zone also decreased in the Sevo group (Fig. 2E-G), suggesting anxiety-like behaviors. In the EPM, impaired locomotor activity was also found in the offspring of the Sevo group indicated by the fewer arm entries compared to the Ctr group (Fig. 2H) while without difference in terms of the ratio of open arm entries to total arm entries and the duration in open arms (Fig. 2I, J). In the TST, the offspring in the Sevo group exhibited a considerably longer duration of immobility than those in the Ctr group (Fig. 2K). Hence, our results suggested that maternal sevoflurane-induced anxiety- and depression-like behaviors in adolescent offspring.

### Maternal sevoflurane exposure impacts interneuron migration in the embryonic cortex

**Fig. 3:**
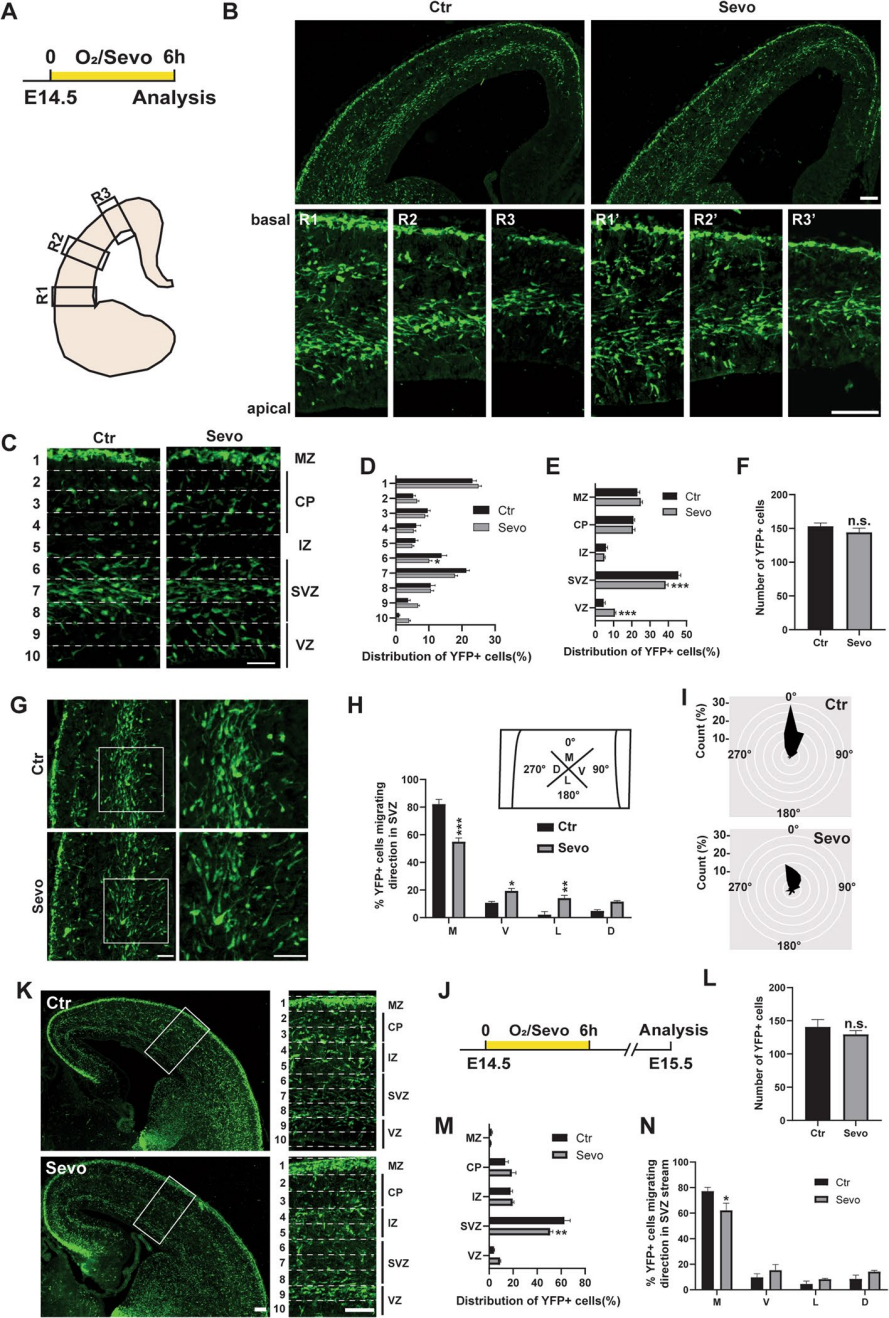
Maternal sevoflurane exposure impacts interneuron migration in the embryonic cortex. **A** Experimental protocols at E14.5. **B** Representative images of sections from fetal brains in Ctr and Sevo groups. Scale bar: 100 μm. **C** Representative images of E14.5 cortical columns. Cortical columns were divided into 10 equal-sized bins, and the number of YFP+ cells were counted in each. Bin 1 and Bin 10 represent the basal and apical side of the dorsal cortex respectively. MZ, marginal zone; CP, cortical plate; IZ, intermediate zone; SVZ, subventricular zone; VZ, ventricular zone. Scale bar: 50 μm. **D** and **E** Radial distribution of YFP+ cells in **C**. Ctr: 12 slices from 4 mice; Sevo: 15 slices from 5 mice. **F** Quantification of YFP+ cells in **C**. Ctr: 12 slices from 4 mice; Sevo: 15 slices from 5 mice. **G** Representative images of fetal dorsal cortex showing the orientations of YFP+ cells. Scale bar: 50 μm. **H** Quantification of the orientations of the leading processes into four quadrants. M, medial; V, ventral; L, lateral; D, dorsal. Ctr: 208 cells from 3 mice; Sevo: 243 cells from 3 mice. **I** The radar plots of the distribution of migrating directions assessed by the angle of the leading process. Angles were grouped into 15° bins and relative percentages were plotted. Each circle represents 5%. Ctr: 208 cells from 3 mice; Sevo: 243 cells from 3 mice. **J** Experimental protocols at E15.5. **K** Representative images of E15.5 cortex. Scale bar: 100 μm. **L** Quantification of YFP+ cells in **K**. Ctr: 9 slices from 3 mice; Sevo: 9 slices from 3 mice. **M** Radial distribution of YFP+ cells in **K**. Ctr: 9 slices from 3 mice; Sevo: 9 slices from 3 mice. **N** Quantification of the orientations of the leading processes into four quadrants. Ctr: 255 cells from 3 mice; Sevo: 193 cells from 3 mice. The data are represented as mean ± SEM. Two-tailed Student’s t-test was performed in **F** and **L**. Two-way ANOVA were performed in **D**, **E**, **H**, **M**, and **N**. *p < 0.05, **p < 0.01, ***p < 0.001, n.s. no significance

Middle pregnancy is the peak of interneuron tangential migration in both humans and rodents [11]. Aberrant migration of inhibitory interneurons can disrupt the inhibitory function and lead to epilepsy [12]. To investigate the effects of maternal sevoflurane exposure on interneuron migration, we analyzed the migratory pattern of cortical interneurons using the Rosa26-EYFP/-; Nkx2.1-Cre mice line, which allowed permanent labeling of MGE-derived interneurons in both embryonic and postnatal stages (Fig. 3A).

At E14.5, most cortical interneurons migrate along two principal routes: a superficial route across the MZ and a deep route that largely overlaps with the subventricular zone (SVZ) (Fig. 3B). However, after the maternal sevoflurane exposure, the migrating interneurons did not show a clear preference for the SVZ route but in a more dispersed manner in radial distribution (Fig. 3C). Quantitative analysis of YFP+ cell showed a lower percentage of interneurons in the SVZ and a higher percentage in the ventricular zone (VZ) in the Sevo group compare to the Ctr group. (Fig. 3D, E). The total number of YFP+ cells was similar between the two groups (Fig. 3F). We then analyzed the migratory direction of individual interneurons, which can be determined by the orientation of the leading process [24]. In the Sevo group, the leading process of migrating interneurons displayed aberrant orientation as fewer cells oriented along the main migration path compared to those in the Ctr group (Fig. 3G–I). Hence, maternal sevoflurane exposure disturbed the tangential migration of cortical interneurons.

To determine whether the effects of sevoflurane on interneuron migration persisted, we analyzed the migratory pattern of cortical interneurons 24 h after sevoflurane treatment (Fig. 3J, K). However, at E15.5, the abnormal radial distribution of cortical interneurons and skewed leading process of YFP+ cells still remained in the Sevo group 24 h after exposure termination (Fig. 3L–N).

### Maternal sevoflurane exposure did not affect the postnatal distribution of cortical interneurons

**Fig. 4:**
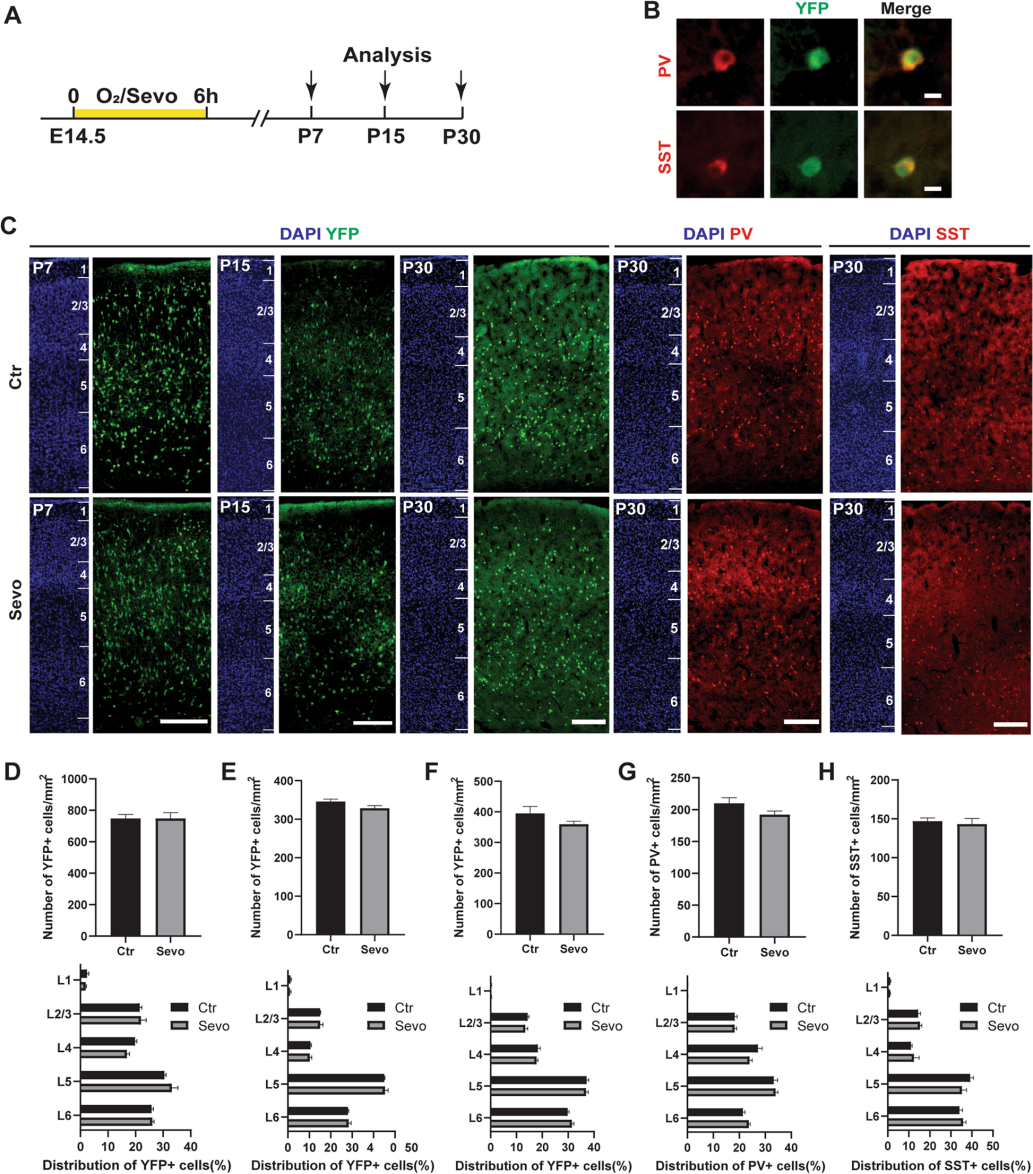
Maternal sevoflurane exposure did not affect the postnatal distribution of cortical interneurons. **A** Experimental protocols. **B** Sample images showing the co-expression of YFP+ neurons with PV and SST. PV, parvalbumin. SST, somatostatin. Scale bar: 10 μm. **C** Representative images showing the laminar distribution of YFP+ cells at P7, , and P30; PV+ and SST+ cells at P30. The crops were organized into 6 layers which could be demarcated by DAPI. Scale bar: 200 μm. **D** Quantity and percentages of YFP+ cells located in different layers at P7. **E** Quantity and percentages of YFP+ cells located in different layers at P15. **F** Quantity and percentages of YFP+ cells located in different layers at P30. **G-H** Quantity and percentages of PV+ and SST+ cells located in different layers at P30. Ctr: 18 slices from 3 mice; Sevo: 18 slices from 3 mice. The data were represented as mean ± SEM. Two-tailed Student’s *t*-test was used for quantity comparisons. Two-way ANOVA was used for analyzing the laminar distribution

Interneuron migration in the rodent cortex continues until the second postnatal week [11]. The embryonic migration defects might affect their postnatal distribution. In mice, more than 30% of the cortical interneurons generated embryonically undergo programmed cell death between the end of the first and the second postnatal week [11]. Therefore, we collected the brains of Rosa26-EYFP/-; Nkx2.1-Cre offspring mice at P7, P15 and P30 (Fig. 4A, C). We found that the quantity and laminar distribution of cortical YFP+ cells were similar between the Ctr and Sevo groups at all the tested timepoints (Fig. 4D–F). To further explore the effect of prenatal sevoflurane exposure on various cortical interneuron subtypes, we co-stained cortical YFP+ cells with PV and SST, markers of the two largest interneuron populations (Fig. 4B, C). The quantity and laminar distribution of cortical PV+ and SST+ cells showed no difference between the Ctr and Sevo groups (Fig. 4G, H). These results suggested that maternal sevoflurane exposure did not affect the postnatal distribution of cortical interneurons in offspring.

### Maternal sevoflurane exposure alters local neural microcircuitry in the cortex of adolescent offspring

**Fig. 5:**
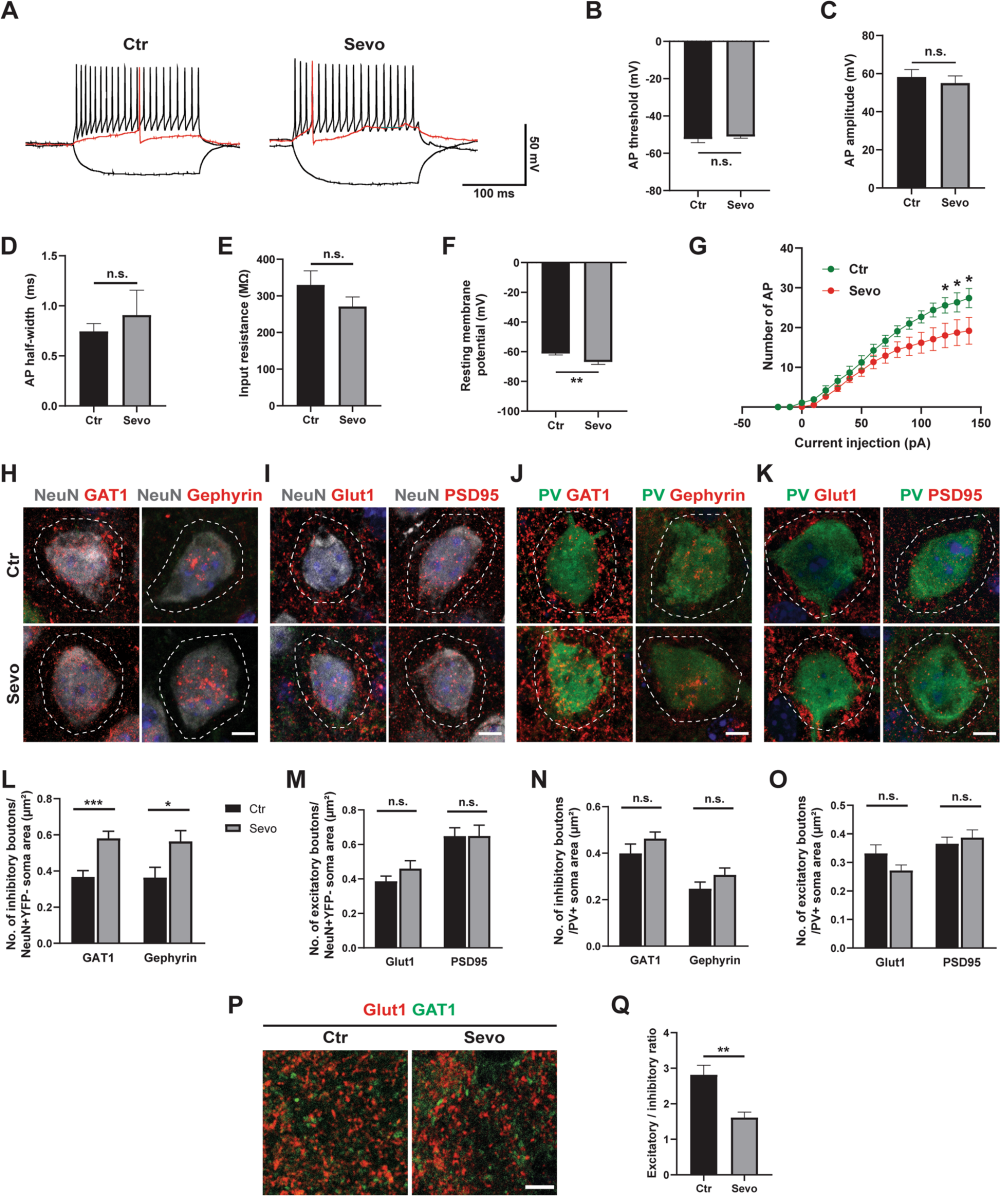
Maternal sevoflurane exposure alters local neural microcircuitry in the cortex of adolescent offspring. **A** Representative traces showing voltage responses of fast-spiking interneurons in Ctr and Sevo groups to step current injections. Red traces indicate the first evoked AP. Ctr: 9 cells from 3 mice; Sevo: 6 cells from 3 mice. **B**–**F** Comparisons about the AP threshold (**B**), AP amplitude (**C**), AP half-width (**D**), input resistance (**E**), and resting membrane potential (**F**) of fast-spiking interneurons. Ctr: 9 neurons from 3 mice; Sevo: 6 neurons from 3 mice. **G** Spike number change of fast-spiking interneurons with different current injections. Ctr: 6 neurons from 3 mice; Sevo: 6 neurons from 3 mice. **H**–**K** The neurons in cortex of P30 mice were co-immunostained with NeuN/GAT1 or NeuN/Gephyrin in (**H**); NeuN/Glut1 or NeuN/PSD95 in (**I**); PV/GAT1 or PV/Gephyrin in (**J**); PV/Glut1 or PV/PSD95 in (**K**); The white dotted circle represented the region of interest for boutons count. Scale bars: 5 μm. **L**–**-O** The differences of excitatory/inhibitory synaptic boutons puncta around the cell soma of NeuN+YFP− (excitatory) (**L**, **M**) or PV+ (inhibitory) (**N**, **O**) neurons between Ctr and Sevo group were analyzed. 7–19 cells from 3 mice per group in each condition. **P** Coronal slices of cortex were co-immunolabeled with Glut1/GAT. Scale bar: 10 μm. **Q** Synaptic puncta were calculated, and the ratio of the integrated density of excitatory and inhibitory puncta in the cortex was shown. 5–7 slices from 3 mice per group. The data are represented as mean ± SEM. Two-tailed Student’s t-test was performed for statistical analysis. *p < 0.05, **p < 0.01, ***p < 0.00, n.s. no significance

Although no difference was detected in the distribution of cortical interneurons between the Ctr and Sevo groups in adolescent mice, the effects on the intrinsic properties of interneurons after maternal sevoflurane exposure remain relatively unexplored. According to recent evidence, deep cortical layers readily generate synchronized epileptiform discharges that propagate into superficial layers and the hippocampus [30]. We therefore analyzed the intrinsic electrophysiological property of layer (L) 5 cortical fast-spiking interneurons, the largest subtype of cortical interneurons that are essential for regulating cortical network excitability and gamma oscillations [31] (Fig. 5A). We found that the threshold, amplitude, and half-width of action potentials (APs) and the input resistance of fast-spiking interneurons were similar between the Ctr and Sevo groups (Fig. 5B–E), indicating that the electrophysiological properties of interneurons in the Sevo group were as matured as it was in the Ctr group, according to a previous description [32]. However, the resting membrane potentials of interneurons in the Sevo group were more hyperpolarized compared to those in the Ctr group (Fig. 5F), which probably prevented fast-spiking interneurons from discharging. Consistent with the hyperpolarized resting membrane potential, fewer artificially induced APs occurred in the interneurons of the Sevo group than in those of the Ctr group (Fig. 5G). Together, these data suggest that maternal sevoflurane exposure impaired the intrinsic electrophysiological properties of cortical fast-spiking interneurons in the adolescent cortex.

Dysfunctional interneurons have been hypothesized to disturb the network stability by influencing the E/I balance of neural circuits [12]. In local neural microcircuits, the E/I balance is indicated by the proportions of excitatory and inhibitory synaptic connections [33]. The structural and functional disturbance of the balance between excitatory and inhibitory synapses is a common pathophysiological feature of epilepsy and multiple seizures [34]. We therefore examined the balance between excitatory and inhibitory synapses by coimmunostaining excitatory (NeuN+YFP−) and fast-spiking inhibitory (PV+) neurons with a series of excitatory and inhibitory pre- and postsynaptic markers, respectively (Fig. 5H–K), according to the methods of a previous study [25]. The density of inhibitory synaptic boutons (GAT+/Gephyrin+) around the somas of excitatory neurons but not around the somas of inhibitory interneurons significantly increased in the deep cortical layers (Fig. 5L, N). The density of excitatory boutons (Glut1+/PSD95+) around the somas of excitatory and inhibitory neurons was comparable between the two groups (Fig. 5M, O). Furthermore, we co-immunostained with Glut1 and GAT1 to analyze the ratio of excitatory versus inhibitory contacts in the cortex (Fig. 5P). A decrease in the E/I ratio was found in the Sevo group compared to the Ctr group (Fig. 5Q). These results suggest that maternal sevoflurane exposure altered the E/I balance in local neural microcircuits by increasing the number of inhibitory synapses.

### The CXCL12/CXCR4 signaling pathway is involved in sevoflurane-induced defects in interneuron migration

Based on the above results, we concluded that maternal sevoflurane exposure disturbed interneuron tangential migration, resulting in a series of defects in adolescent mice. To investigate the underlying mechanism, we explored a potential target that plays a pivotal role in orchestrating the organization of cortical interneuron migration in the fetal brain.

**Fig. 6:**
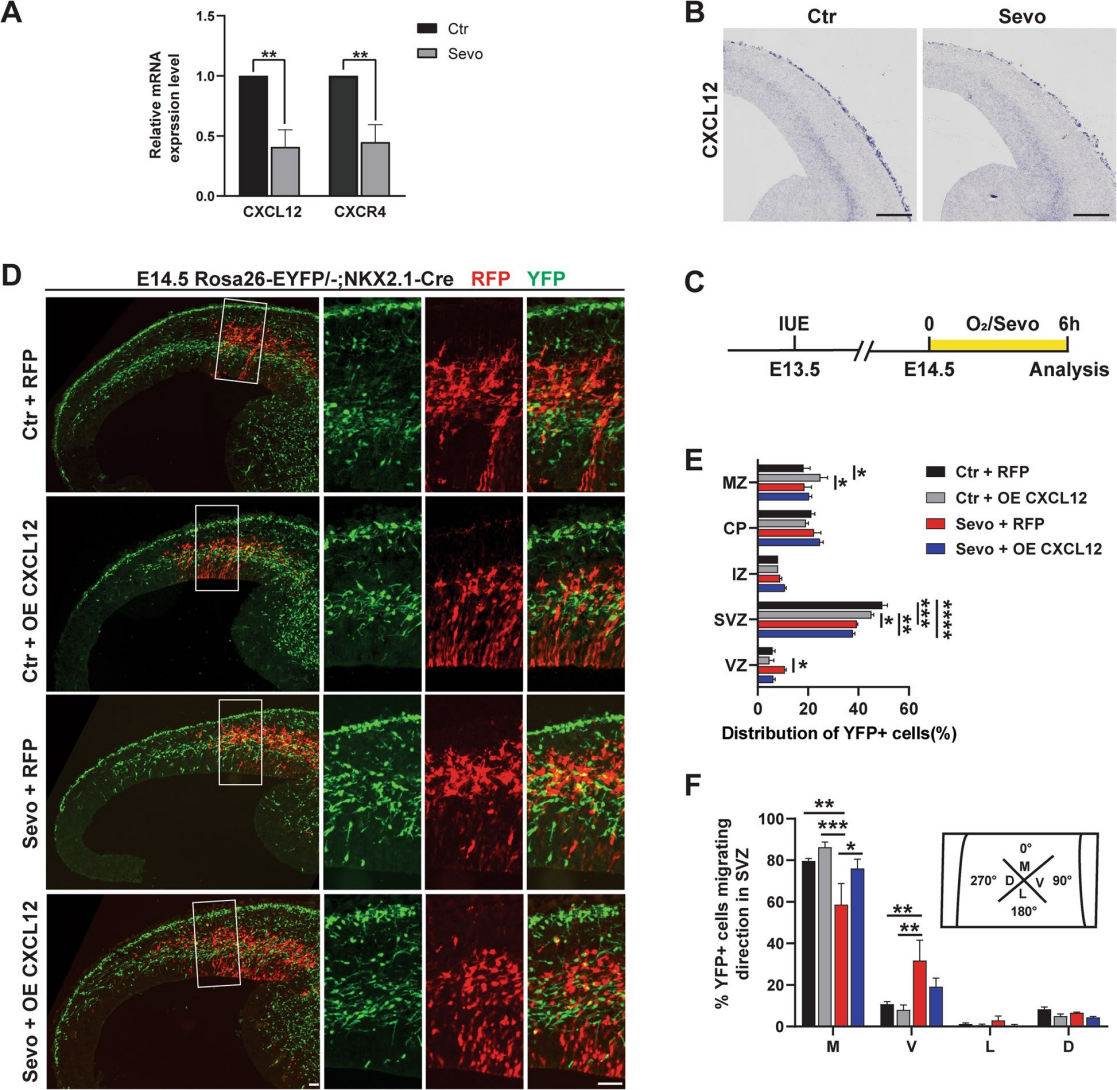
The CXCL12/CXCR4 signaling pathway is involved in sevoflurane-induced defects in interneuron migration. **A** Q-PCR analysis. Ctr: *n*=3; Sevo: *n*=3. **B** Representative ISH images. Scale bar: 200 μm. **C** Experimental protocols for overexpressing CXCL12 in the embryonic cortex by conducting IUE at E13.5. **D** Representative images of sections from E14.5 embryonic brains in the Ctr group, Sevo group, CXCL12+RFP Sevo group (offspring hemispheres electroporated with CXCL12 and RFP at E13.5 and exposed to sevoflurane at E14.5), and RFP Sevo group (offspring hemispheres electroporated with RFP without CXCL12 at E13.5 and exposed to sevoflurane at E14.5). Scale bar: 50 μm. **E** Radial distribution of YFP+ cells. **F** Quantification of leading process orientation into four quadrants. M, medial; V, ventral; L, lateral; D, dorsal. Ctr: 129 cells from 3 mice; Sevo: 118 cells from 3 mice; CXCL12+RFP Sevo: 154 cells from 3 mice; RFP Sevo: 134 cells from 3 mice. The data are represented as mean ± SEM. Two-tailed Student’s *t*-test (**A**) and one-way ANOVA (**E**, **F**) were performed for statistical analysis. *p < 0.05, **p < 0.01, ***p < 0.001

In the cortex, C-X-C motif chemokine ligand-12 (CXCL12; also known as stromal cell-derived factor 1, SDF1) is secreted by the meninges and intermediate progenitor cells in the SVZ. It attracts CXCR4-expressing interneurons, maintaining these interneurons in the MZ and SVZ [35]. Previous studies have shown that reduced expression of CXCL12 and its receptors may contribute to disorganized tangential migration [36, 37]. Therefore, we examined the mRNA expression of CXCL12/CXCR4 at E14.5 after maternal sevoflurane exposure by q-PCR and ISH. The results showed that the CXCL12/CXCR4 signaling pathway was downregulated after anesthesia exposure (Fig. 6A, B).

To further confirm the role of the CXCL12/CXCR4 signaling pathway in the effect of maternal sevoflurane exposure, we electroporated a CXCL12 expression vector labeled by RFP into the lateral neocortical ventricular zone at E13.5 (Fig. 6C; Additional file 2: Fig. S1A). We verified the overexpression of CXCL12 after 1 day (at E14.5) by immunofluorescence (Additional file 2: Fig. S1B). The overexpression of CXCL12 in the Sevo + OE CXCL12 group attenuated the elevated proportion of cortical interneurons in VZ and abnormal migratory orientation induced by sevoflurane exposure (Fig. 6D–F). These data show that maternal sevoflurane exposure impairs interneuron migration through the CXCL12/CXCR4 signaling pathway.

### Overexpression of CXCL12 rescues the increased epilepsy susceptibility and ameliorates abnormal behaviors in Sevo group

**Fig. 7:**
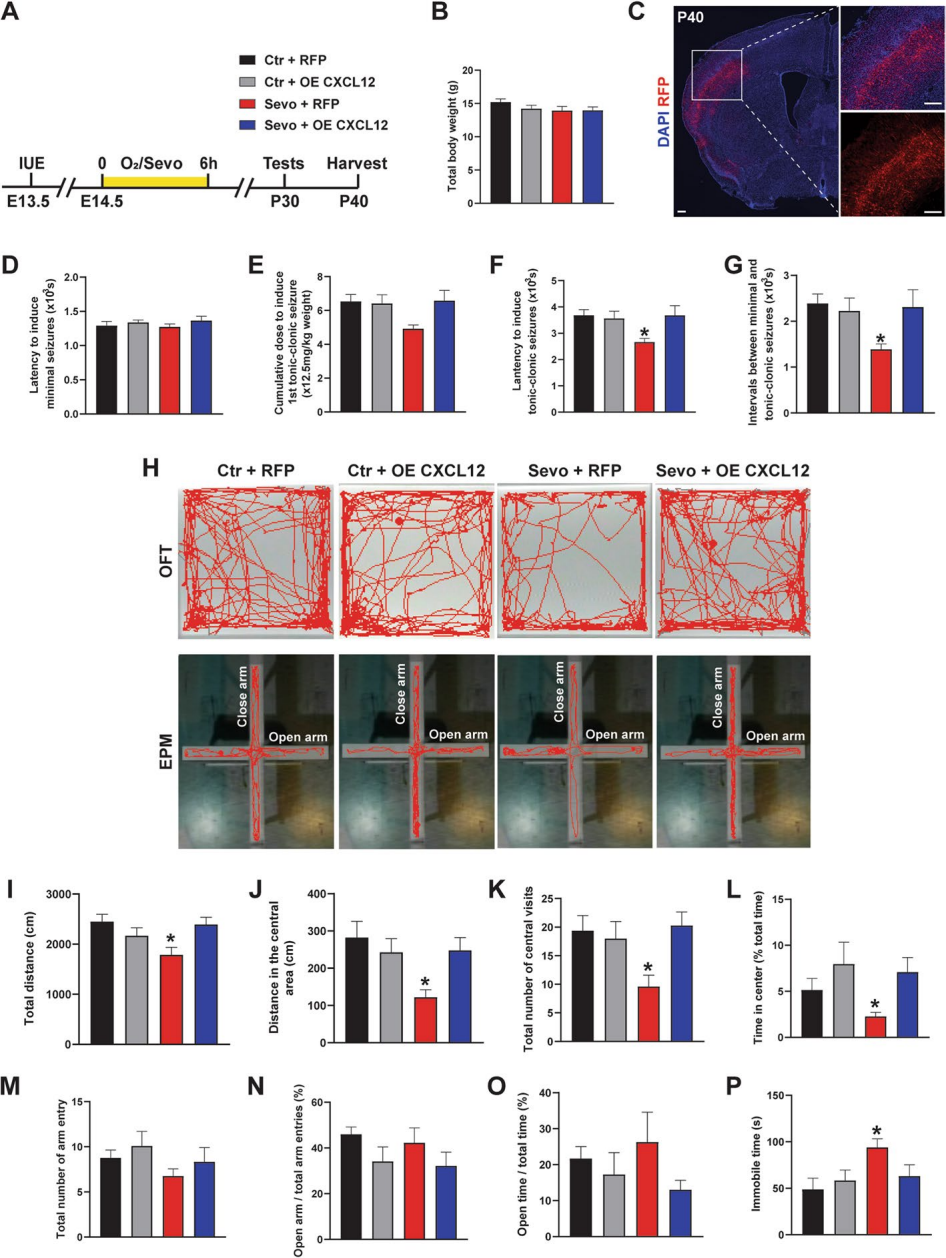
The overexpression of CXCL12 rescues the increased epilepsy susceptibility and ameliorates abnormal behaviors in adolescent offspring. **A** Experimental design. **B** Quantification of total body weight in the four groups. **C** Representative images showing the expression of RFP in the cortex which indicated the electroporation. Scale bar: 250 μm. **D** Comparisons about the latency to induce minimal seizures. **E** Comparisons about the cumulative doses required to induce the 1st tonic-clonic seizures. Ctr+RFP vs Sevo+RFP: *p*=0.0697. **F** Comparisons about the latency required to induce the 1st tonic-clonic seizures. Ctr+RFP vs Sevo+RFP: *p*=0.0413. **G** Comparisons about the intervals between minimal and tonic-clonic seizures. Ctr+RFP vs Sevo+RFP: *p*=0.0430. **H** The representative traces in the OFT within 10 min and the representative traces in the EPM in 5 min. **I-L** Comparisons about the total distance traveled (**I**), the central distance traveled (**J**), the number of central visits (**K**), and the percentage of central time (**L**). Ctr+RFP vs Sevo+RFP: *p*=0.0153, 0.0118, 0.0413, and 0.0463, respectively. **M**–**O** Comparisons about the total number of arm entries (**M**), the ratio of open/total arm entries (**N**), and the ratio of the duration in open arm/total time (**O**). No significance between the groups. **P** The immobile time in the TST. Ctr+RFP vs Sevo+RFP: *p*=0.0316. Ctr + RFP: *n*=13, Ctr + OE CXCL12: *n*=12, Sevo + RFP: *n*=12, Sevo + OE CXCL12: *n*=12. The data are represented as mean ± SEM. One-way ANOVA was performed for statistical analysis. *p < 0.05

Next, we examined whether overexpression of CXCL12 could rescue the increased epilepsy susceptibility and ameliorate abnormal behaviors in adolescent offspring (Fig. 7A, B). The total body weight had no difference between groups (Fig. 7C). In the epilepsy susceptibility test, overexpression of CXCL12 prolonged the latency and increased cumulative doses of PTZ to induce tonic-clonic seizures (Fig. 7D–G), rescuing the increased epilepsy susceptibility caused by prenatal sevoflurane exposure. In the OFT, the mice in Sevo + OE CXCL12 group showed a longer total distance traveled, more central visits, longer central distance traveled and duration time compared to the offspring in the Sevo + RFP group (Fig. 7H–L), ameliorating anxiety-like behaviors. While in the EPM test, we did not find the difference between the groups (Fig. 7H, M–O). In the TST, overexpression of CXCL12 reversed the extended duration of immobility (Fig. 7P), ameliorating depression-like behavior. Taken together, overexpression of CXCL12 can rescue the increased epilepsy susceptibility and ameliorate abnormal behaviors in Sevo group offspring mice.

### Sevoflurane-induced defects in adolescence are temporary rather than long-lasting

It has been reported that some features of interneurons such as synaptic connections undergo reversible changes in the adult brain to mediate the adaptation to the environment [11]. Hence, we examined whether the increased epilepsy susceptibility and behavioral alterations during adolescence would persist into adulthood (Additional file 3: Fig. S2A). At P60, equivalent to adulthood in humans [28], the performance of Sevo group mice was similar to those in the Ctr group in the epilepsy susceptibility assay and behavioral tests (Additional file 3: Fig. S2B–M). In addition, the density and proportion of inhibitory synapses in the deep cortical layer were also comparable between the two groups (Additional file 3: Fig. S2N–Q). Taken together, these results suggested that the defects observed in adolescence were temporary.

## Discussion

Our study demonstrated that maternal sevoflurane exposure increased epilepsy susceptibility and induced anxiety- and depression-like behaviors in offspring during adolescence but not in adulthood. Maternal sevoflurane exposure impaired the migration of MGE-derived interneurons during the peak of neurogenesis in embryonic brains and changed their postnatal electrophysiological properties. Furthermore, the CXCL12/CXCR4 signaling pathway played an important role in the migration defects of interneurons which may contribute to the increased epilepsy susceptibility in adolescent offspring. Taken together, our findings show that exposure to maternal anesthesia induced deficits in interneuron development and long-term neuronal dysfunction.

We reported the increased epilepsy susceptibility in adolescent offspring by maternal sevoflurane exposure during middle pregnancy for the first time (Fig. 1). Until now, a direct link between epilepsy susceptibility and anesthetic exposure during brain development has not been explored. In this study, adolescent mice with prenatal sevoflurane exposure did not exhibit spontaneous seizure activity, but they had a higher seizure susceptibility indicated by a lower threshold of tonic-clonic seizures induced by PTZ (Fig. 1D–H). This finding is of great clinical importance and highlights that the drugs with seizure-inducing side effects should be administered with caution to children and adolescents who experienced prenatal anesthesia exposure. For example, the incidence of attention-deficit hyperactivity disorder (ADHD) is approximately 3–5% in children worldwide [38]. Methylphenidate is the first-line medication for ~90% of children with ADHD [39, 40]. However, a large population-based observational study reported that the incidence of seizures in children with ADHD was higher in the period immediately after the start of methylphenidate treatment [41]. Thus, our results suggest that medication such as methylphenidate should be used with great caution in children with ADHD and a history of prenatal anesthesia exposure.

Additionally, we found that the offspring under prenatal sevoflurane exposure displayed anxiety- and depression-like behaviors at the adolescent stage (Fig. 2). Sevoflurane exposure has been reported to induce anxiety- and depression-like behaviors through different mechanisms including activating microglia, interfering with metabolism pathway and disrupting E/I balance in the neural network [42–44]. Our study confirms that maternal sevoflurane exposure increases the seizure susceptibility as well as induces abnormal behaviors in adolescent offspring.

A higher risk of epilepsy is strongly associated with the dysfunction or loss of cortical interneurons [13]. Our results showed that maternal sevoflurane exposure induced defective migration of MGE-derived interneurons in the embryonic cortex at the peak of neurogenesis (Fig. 3). Neuronal migration is one of the key components of brain development; it commences in the prenatal period and continues into postnatal development. Disruption to neural migration is strongly associated with brain dysfunction [10]. Our data showed that the orientations of the leading processes of interneurons were skewed under sevoflurane (Fig. 3G–I), resulting in deviation of migration from the SVZ path (Fig. 3C–E). Thus, we provide the evidence that GABAergic inhibitory interneurons in the fetal cortex are affected by maternal anesthesia exposure.

Nevertheless, in our study, maternal sevoflurane exposure did not alter the final distribution of cortical interneurons (Fig. 4). Previous studies have reported mixed results on this topic. In mice, maternal exposure to 4% sevoflurane for 3 h at E14.5 significantly decreased the number of interneurons, especially PV+ interneurons, in the offspring L2/3 entorhinal cortex at P30 [45]. In contrast, exposure to 3.0% sevoflurane resulted in a significant increase in PV + interneurons in the medial prefrontal cortex at P15, P30, and P60 [44]. Thus, differences in anesthetic concentrations and observed brain regions might explain the discrepancies in the results. In basic research, emerging evidence has indicated that high concentrations of sevoflurane anesthesia inhibited proliferation and induced neuronal apoptosis in vivo and in vitro [46, 47]. Additionally, in clinical situations, inhaling high concentrations of sevoflurane causes circulatory changes in the mother and directly affects the blood supply and health of the fetus [48]. Therefore, we used 2.5% sevoflurane, the MAC of sevoflurane for rodents [22], with our pregnant mice, to avoid the extreme disadvantages found with high concentrations of sevoflurane.

After the cell death period, the number of interneurons is maintained throughout life, and functional integration continues up to 3–4 weeks postnatally in rodents, paralleled by substantial alterations in the signaling properties of the cells [49]. Here, we showed that prenatal sevoflurane exposure induced a hyperpolarized resting membrane potential as well as fewer evoked APs in fast-spiking interneurons in the adolescent offspring cortex (Fig. 5F, G). The loss or dysfunction of interneurons has been reported to induce the formation of synapses, which is likely a compensatory response that maintains the E/I balance of neural circuits but may also contribute to network destabilization [50, 51]. In our study, we observed an increase in inhibitory synapses after maternal sevoflurane exposure (Fig. 5H, L, P, Q). Therefore, alterations of the intrinsic electrophysiological properties and local neural microcircuitry induced by prenatal sevoflurane exposure might serve as the electrophysiological and histological basis of network destabilization, leading to increased epilepsy susceptibility and abnormal behaviors in adolescence.

Several secreted guidance factors that direct interneurons along their migratory paths have been identified, including CXCL12 [11]. In the central nervous system, CXCL12 binds to its receptor CXCR4 and activates a series of downstream signaling pathways, such as the Wnt/β-catenin, PI3/Akt, and JAK/STAT pathways, to regulate neurogenesis, including neural migration [52]. We confirmed that the CXCL12/CXCR4 signaling pathway was downregulated in the fetal cortex of mice in the Sevo group with q-PCR and ISH (Fig. 6A, B). Previous studies have demonstrated that the loss of CXCL12/CXCR4 resulted in deviations of migrating interneurons from their tangential trajectory and premature invasion of the cortical plate from the SVZ [36, 37], consistent with our results. In addition, SDV1a, a CXCL12 analog chemically created by Lee et al., elicited extensive and persistent human neural stem cell migration and distribution without inducing host inflammation or other adverse effects [53]. In our study, we utilized IUE to overexpress CXCL12, which was able to reverse the disturbed migration induced by sevoflurane exposure (Fig. 6D–F). More importantly, the overexpression of CXCL12 mitigates the increased epilepsy susceptibility and abnormal behaviors in adolescent offspring (Fig. 7). Therefore, our research illustrates a new molecular pathway underlying the disturbed fetal neurogenesis induced by maternal anesthetic exposure.

Notably, the increased epilepsy susceptibility, abnormal behaviors, and alterations of cortical microcircuitry induced by maternal sevoflurane exposure were observed in adolescence but not in adulthood in the offspring (Additional file 3: Fig. S2). Adolescence is a time of physiological and psychosocial change and is a sensitive period for the development of higher-order cognition due to enhanced plasticity of cortical circuits. In offspring, the overproduction of synapses during postnatal development contributes to enhanced plasticity by providing an excess of synapses that are then pruned during early adolescence [54]. This evidence partially explains our observations of normal synapse arrangements in adulthood. Additionally, these differences might indicate the possibility of a self-recovery mechanism. Further study is needed to elucidate the mechanisms underlying this recovery and identify potential treatments for certain neurodevelopment disorders.

The neurotoxicity caused by anesthetic exposure is primarily dependent on the concentration of the anesthetic and the duration of exposure. In our study, prenatal 2.5% sevoflurane exposure for 6 h increased epilepsy susceptibility and induced abnormal behaviors in adolescent mice with intact terminal quantity and distribution of interneurons. Previous studies reveal that under higher concentration or multiple exposure, sevoflurane influences the quantity and distribution of interneurons and damages brain functions in offspring mice, which could even last to adulthood [44, 55]. As to the maternal exposure, different trimesters might show different effects. In our study, the sevoflurane exposure was conducted in the second trimester. It has been reported that maternal anesthesia during the first and third trimesters also altered brain structure but in different ways with increased risks of adverse pregnancy such as spontaneous abortion and lower birth weight [56, 57].

Although in this study we focused on cortical interneurons, hippocampus, and striatal GABAergic interneurons are also likely to be affected by maternal sevoflurane exposure since they also undergo migration at E14.5 [11]. A disruption of these neuronal populations could impact the maturation of GABAergic interneurons and hamper their functional integration into cortical circuits, thus compounding the overall effect of sevoflurane on the development of GABAergic interneurons.

## Conclusions

We demonstrated that maternal sevoflurane exposure at the peak of neurogenesis in offspring increased epilepsy susceptibility and induced anxiety- and depression-like behaviors in adolescence. These findings emphasize the need for caution when prescribing drugs with seizure-inducing side effects to children with a history of maternal anesthesia exposure, such as the use of methylphenidate to treat children with ADHD. In addition, we identified and verified CXCL12/CXCR4 as a targeted signaling pathway through which sevoflurane disrupts the migration of interneurons and impaired the long-term neuronal functions.

### Supplementary Information

**Additional file 1 **: **Table S1**. Antibodies and primers.

**Additional file 2 **: **Fig. S1**. The overexpression of CXCXL12 through IUE in embryonic cortex. A Plasmids expressing CXCL12 and pCAGEN (as a control) were electroporated into cortex in utero. B Representative images showing the overexpression of CXCL12 in embryonic cortex by IUE. Scale bar: 200 μm.

**Additional file 3 **: **Fig. S2**. Sevoflurane-induced defects in adolescence are temporary rather than long-lasting. A Experimental protocols at P60. B-E Analysis of epilepsy susceptibility. Ctr: *n*=20; Sevo: *n*=16. F-L Results of OFT and EPM. Ctr: *n*=14; Sevo: *n*=14. M The immobile time in TST. Ctr: *n*=17; Sevo: *n*=20. N The excitatory neurons (NeuN+YFP-) were coimmunostained with GAT/Gephyrin. Scale bars: 5 μm. O Analysis of inhibitory synaptic boutons puncta around the soma of excitatory neurons. 8–16 slices from 3 mice per group. P Coronal slices of cortex were co-immunolabeled with Glut1/GAT. Scale bar: 10 μm. Q The ratio of the integrated density of excitatory and inhibitory puncta in cortex. 8–16 slices from 3 mice per group. The data are represented as mean ± SEM. Two-tailed Student’s t-test was performed for statistical analysis. n.s. no significance.

## Data Availability

All data generated or analyzed during this study are included in this published article.

## Abbreviations

AP: Action potential
ACSF: Artificial cerebral spinal fluid
ADHD: Attention-deficit hyperactivity disorder
Ctr: Control
L: Cortical layer
CXCL12: C-X-C motif chemokine ligand-12
CXCR4: C-X-C-chemokine receptor 4
DIG: Digoxigenin
EPM: Elevated plus maze
E: Embryonic day
E/I: Excitatory/inhibitory
ISH: In situ hybridization
IUE: In utero electroporation
MZ: Marginal zone
MGE: Medial ganglionic eminence
MAC: Minimum alveolar concentration
OFT: Open field test
PFA: Paraformaldehyde
PV: Parvalbumin
PTZ: Pentylenetetrazole
PBS: Phosphate buffered saline
P: Postnatal day
Sevo: Sevoflurane
SST: Somatostatin
SEM: Standard error of the mean
SVZ: Subventricular zone
TST: Tail suspension test
VZ: Ventricular zone
YFP: Yellow fluorescent protein
