# Supplementary material for: Maternal sevoflurane exposure increases the epilepsy susceptibility of adolescent offspring by interrupting interneuron development
Source: BMC Med. 2023 Dec 21;21:510. doi: 10.1186/s12916-023-03210-0 (PMC10740307; doi:10.1186/s12916-023-03210-0)
Supplement: Supplementary file 1 — Additional file 1 : Table S1. Antibodies and primers. [file 12916_2023_3210_MOESM1_ESM.docx]

Table S1

| Antibodies | | | | |
| --- | --- | --- | --- | --- |
| Name | | Source | Identifier | Dilution |
| Chicken anti-GFP | | Abcam | Cat# ab13970, RRID: AB_300798 | 1:2000 |
| Goat anti-parvalbumin | | Abcam | Cat# ab11427, RRID: AB_298032 | 1:2000 |
| Rabbit anti-somatostatin | | Abcam | Cat# ab64053, RRID: AB_1143012 | 1:800 |
| Mouse anti-NeuN | | Sigma-Aldrich | Cat# MAB377, RRID: AB_2298772 | 1:500 |
| Rabbit anti GAT1 | | Abcam | Cat# ab259971, RRID: AB_3068552 | 1:250 |
| Rabbit anti-NeuN | | Abways | Cat# CY5515, RRID: AB_3068554 | 1:800 |
| Mouse anti-Gephyrin | | Synaptic Systems | Cat# 147011, RRID: AB_887717 | 1:500 |
| Rabbit anti-Glut1 | | Abcam | Cat# ab272913, RRID: AB_3068553 | 1:1000 |
| Rabbit anti-PSD95 | | Abcam | Cat# ab18258, RRID: AB_444362 | 1:2000 |
| Guinea pig anti-VGAT | | Synaptic Systems | Cat# 131004, RRID: AB_887873 | 1:250 |
| Rabbit anti-RFP | | Rockland | Cat# 600-401-379, RRID: AB_2209751 | 1:1000 |
| Alexa Fluor 488 goat anti-chicken | | Thermo Fisher Scientific | Cat# A11039, RRID: AB_2534096 | 1:800 |
| Alexa Fluor 488 goat anti-guinea pig | | Thermo Fisher Scientific | Cat# A1073, RRID: AB_2534117 | 1:800 |
| Alexa Fluor 488 donkey anti-chicken | | Jackson ImunoResearch | Cat# 703-545-155, RRID: AB_2340375 | 1:800 |
| Alexa Fluor 568 goat anti-rabbit | | Thermo Fisher Scientific | Cat# A11036, RRID: AB_10563566 | 1:800 |
| Alexa Fluor 568 goat anti-mouse | | Thermo Fisher Scientific | Cat# A11031, RRID: AB_144696 | 1:800 |
| Alexa Fluor 568 donkey anti-goat | | Thermo Fisher Scientific | Cat# A11057, RRID: AB_2534104 | 1:800 |
| Alexa Fluor 647 goat anti-mouse | | Thermo Fisher Scientific | Cat# A21236, RRID: AB_2535805 | 1:800 |
| Alexa Fluor 647 goat anti-rabbit | | Thermo Fisher Scientific | Cat# A21245, RRID: AB_2535813 | 1:800 |
| Primers | | | | |
| CXCL12 q-PCR primers | Forward sequence: TGCATCAGTGACGGTAAACCA  Reverse sequence: TTCTTCAGCCGTGCAACAATC | | | |
| CXCR4 q-PCR primers | Forward sequence: GACTGGCATAGTCGGCAATG  Reverse sequence: AGAAGGGGAGTGTGATGACAAA | | | |
| CXCL12 ISH probe primers | Forward sequence: CTGCAGGAATTCGATAATTTCGGGTCAATGCACAC  Reverse sequence: ATCGATAAGCTTGATCACTTTCACTCTCGGTCCAC | | | |
| CXCL12 clone primers | Forward sequence: TTTGGCAAAGAATTCATGGACGCCAAGGTCGTCGC  Reverse sequence: CGCGATATCCTCGAGGTTTTTCCTTTTCTGGGCAG | | | |
