# Supplementary figures and images for: Maternal sevoflurane exposure increases the epilepsy susceptibility of adolescent offspring by interrupting interneuron development
Source: BMC Med. 2023 Dec 21;21:510. doi: 10.1186/s12916-023-03210-0 (PMC10740307; doi:10.1186/s12916-023-03210-0)

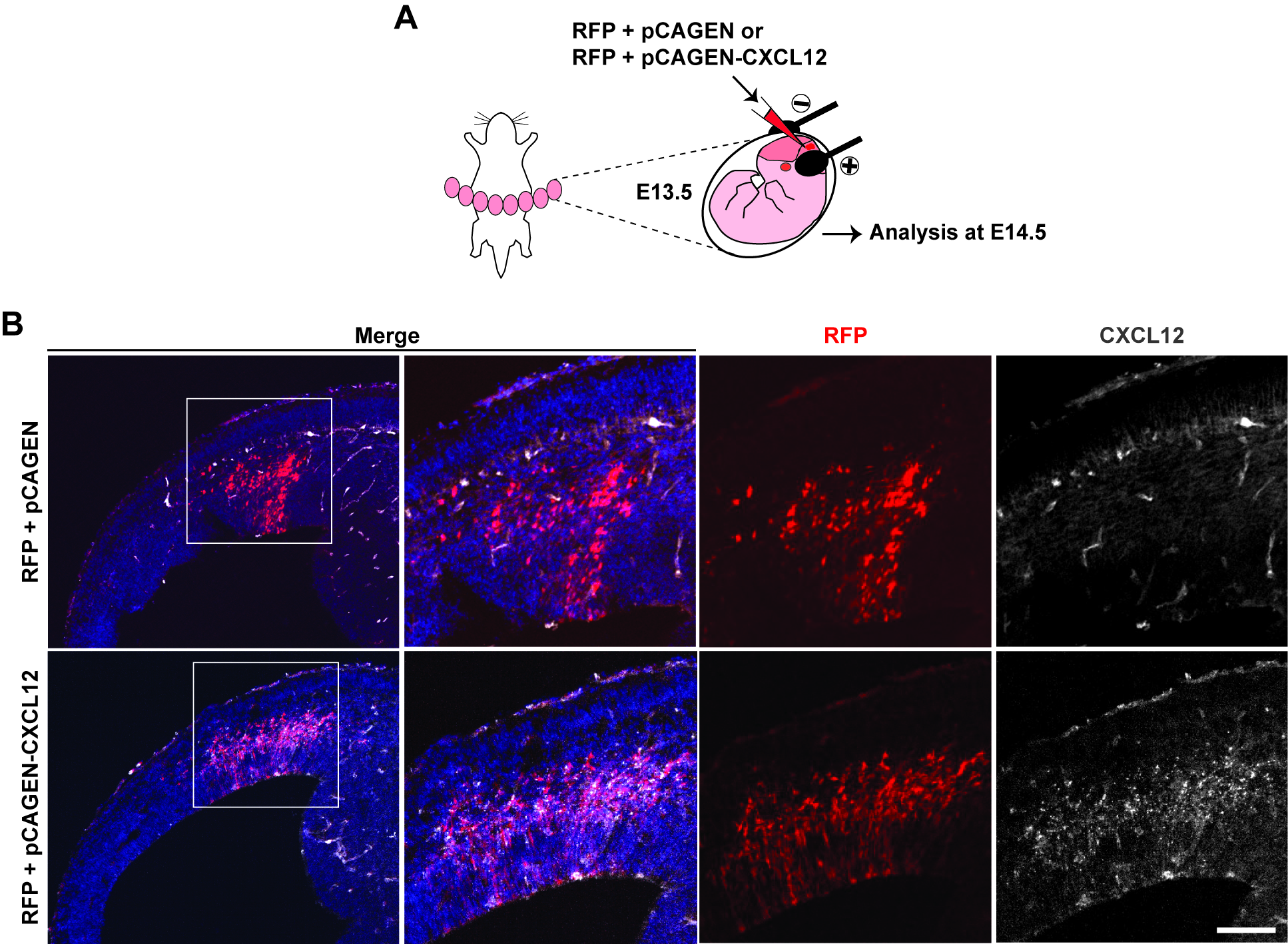

Supplement: Supplementary file 2 — Additional file 2 : Fig. S1. The overexpression of CXCXL12 through IUE in embryonic cortex. A Plasmids expressing CXCL12 and pCAGEN (as a control) were electroporated into cortex in utero. B Representative images showing the overexpression of CXCL12 in embryonic cortex by IUE. Scale bar: 200 μm. [file 12916_2023_3210_MOESM2_ESM.tif]

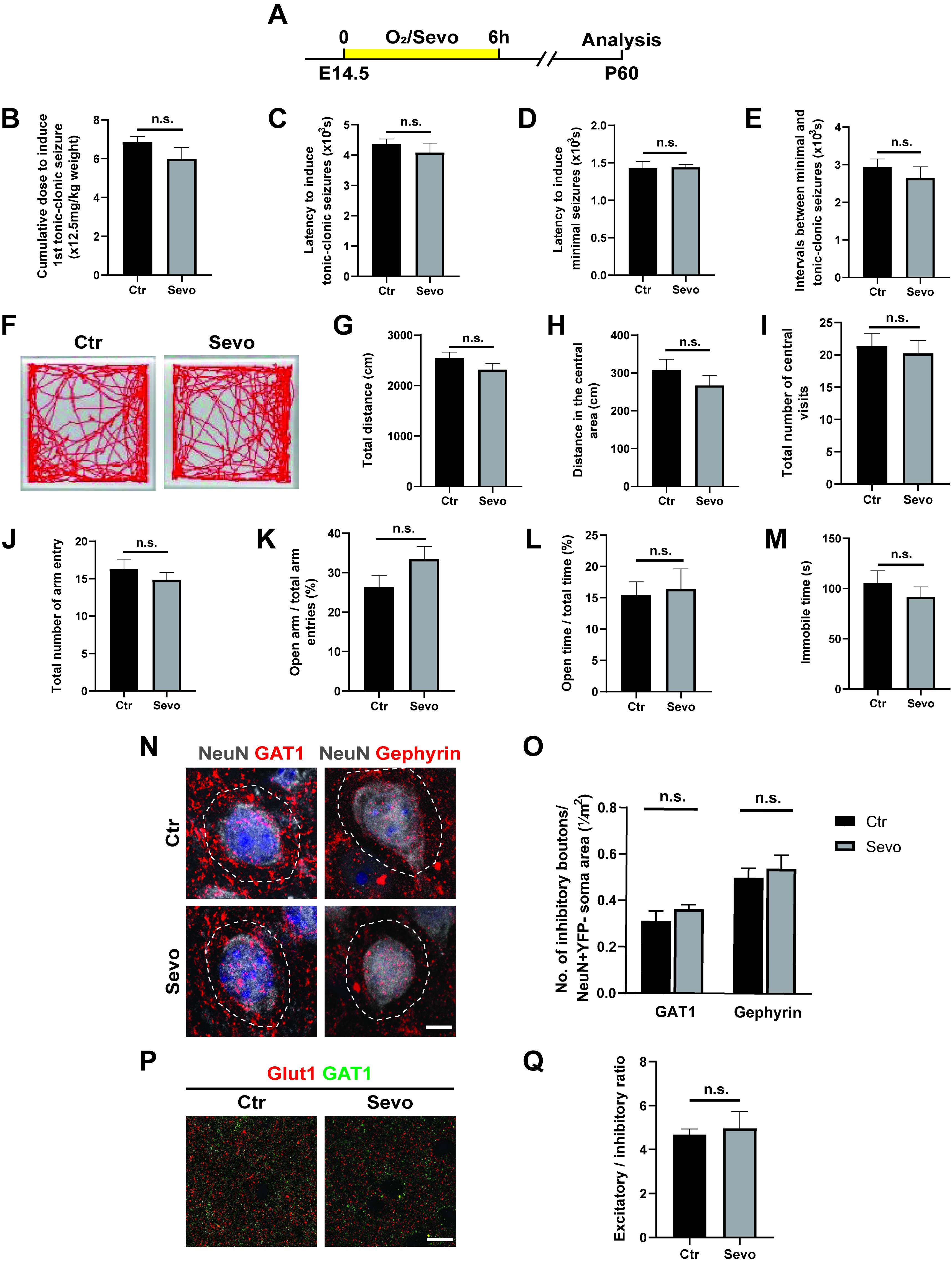

Supplement: Supplementary file 3 — Additional file 3 : Fig. S2. Sevoflurane-induced defects in adolescence are temporary rather than long-lasting. A Experimental protocols at P60. B-E Analysis of epilepsy susceptibility. Ctr: n=20; Sevo: n=16. F-L Results of OFT and EPM. Ctr: n=14; Sevo: n=14. M The immobile time in TST. Ctr: n=17; Sevo: n=20. N The excitatory neurons (NeuN+YFP-) were coimmunostained with GAT/Gephyrin. Scale bars: 5 μm. O Analysis of inhibitory synaptic boutons puncta around the soma of excitatory neurons. 8–16 slices from 3 mice per group. P Coronal slices of cortex were co-immunolabeled with Glut1/GAT. Scale bar: 10 μm. Q The ratio of the integrated density of excitatory and inhibitory puncta in cortex. 8–16 slices from 3 mice per group. The data are represented as mean ± SEM. Two-tailed Student’s t-test was performed for statistical analysis. n.s. no significance. [file 12916_2023_3210_MOESM3_ESM.tif]
